# Intratumoural production of TNFα by bacteria mediates cancer therapy

**DOI:** 10.1371/journal.pone.0180034

**Published:** 2017-06-29

**Authors:** Carola Murphy, Elizabeth Rettedal, Panos Lehouritis, Ciarán Devoy, Mark Tangney

**Affiliations:** 1 Cork Cancer Research Centre, University College Cork, Cork, Ireland; 2 SynBioCentre, University College Cork, Cork, Ireland; 3 APC Microbiome Institute, University College Cork, Cork, Ireland; Instituto Butantan, BRAZIL

## Abstract

Systemic administration of the highly potent anticancer therapeutic, tumour necrosis factor alpha (TNFα) induces high levels of toxicity and is responsible for serious side effects. Consequently, tumour targeting is required in order to confine this toxicity within the locality of the tumour. Bacteria have a natural capacity to grow within tumours and deliver therapeutic molecules in a controlled fashion. The non-pathogenic *E*. *coli* strain MG1655 was investigated as a tumour targeting system in order to produce TNFα specifically within murine tumours. *In vivo* bioluminescence imaging studies and *ex vivo* immunofluorescence analysis demonstrated rapid targeting dynamics and prolonged survival, replication and spread of this bacterial platform within tumours. An engineered TNFα producing construct deployed in mouse models via either intra-tumoural (i.t.) or intravenous (i.v.) administration facilitated robust TNFα production, as evidenced by ELISA of tumour extracts. Tumour growth was impeded in three subcutaneous murine tumour models (CT26 colon, RENCA renal, and TRAMP prostate) as evidenced by tumour volume and survival analyses. A pattern of pro-inflammatory cytokine induction was observed in tumours of treated mice vs. controls. Mice remained healthy throughout experiments. This study indicates the therapeutic efficacy and safety of TNFα expressing bacteria *in vivo*, highlighting the potential of non-pathogenic bacteria as a platform for restricting the activity of highly potent cancer agents to tumours.

## Introduction

The efficacy of current anti-cancer small drug chemotherapeutics is limited because of the narrow therapeutic index inherent in most of the drugs employed to treat cancer which leads to systemic damage of healthy tissue and side effects upon treatment. For this reason, alternative therapies for the treatment of cancer that aim to localize the therapeutic agent to the site of the tumour are been investigated. TNFα was identified in 1975 when it was discovered that a substance from the sera of animals that were challenged with BCG and endotoxin could kill mouse cells *in vitro* and induce haemorrhagic necrosis of transplantable mouse tumours *in vivo* [1]. Subsequently, TNFα was investigated as a therapeutic agent for cancer treatment. However, due to severe systemic toxicity it was soon abandoned for systemic use, only to be revisited later in the settings of isolated limb perfusion to treat inoperable cancer [2]. The inherent high level of toxicity of TNFα poses health risks, and therefore it is essential that if it is to be used for treating cancer it must be confined to the tumour site in a highly controlled manner. Biological vehicles have been examined for this purpose in the context of cancer gene therapy, and TNFα delivery by viruses such as adeno-associated virus [3] or adenoviruses have shown promise. TNFerade is a serotype 5 adenovirus that expresses TNFα under the control of the early growth response gene (egr-1) promoter that responds to radiation, which has been examined in Phase 3 clinical trials for advanced prostate cancer [4–7]. In this approach, while the biological delivery vehicle is not confined to tumours, TNFα production is restricted via physically targeted radiation induction of the egr-1 promoter to express the TNFα transgene.

Bacteria represent another class of cancer gene therapy vector that have an established safety profile and track record of facilitating protein production within tumours [8, 9]. Unlike viral vectors, which induce agent production via transduction of cells followed by host cell expression of the delivered transgene, bacteria provide the option of host cell production (through employment of an invasive strain–aka ‘bactofection’ [10, 11]) or the bacterium can express the agent directly. For the latter, non-pathogenic strains of bacteria may be used (e.g. probiotics), increasing the safety profile of the platform [12]. Bacteria came to be investigated as cancer therapeutic agents due to their natural ability to grow within tumours [13]. The primary factors believed to be responsible for tumour-selective survival and replication involve tissue traits unique to tumours; irregular leaky vasculature permits bacterial entry to tissue, local immune suppression allows the bacteria to ‘hide’ from the immune system, tumour cell necrosis provides a rich nutrient supply, and anaerobic/facultative-anaerobic bacteria grow well in the hypoxic tissue (unique to tumours). Bacteria have a number of other advantages over viral vectors as delivery vehicles: they have a large genome capable of carrying large therapeutic genes or plasmids; they can be engineered in a highly sophisticated fashion; many are motile and can penetrate deep within the tumour; and, if needed, they can be eliminated with antibiotics. Various cytokines have been delivered to tumours by bacteria in the past with varying degrees of success. For example, *Salmonella* strains have been used in conjunction with IL-12 [14], IL-4 and IL-18 [15][16], TRAIL [17] and FAS ligand [18] and some *Clostridium* strains with TNFα [19] and IL-2 [20].

In this study, we demonstrate the utility of the naturally non-pathogenic *E*. *coli* MG1655 as a platform for safe and effective *in situ* biomolecule production, and the capacity to improve the safety profile of promising anticancer agents through employment of this platform.

## Material and methods

### Cell lines

RENCA (mouse renal carcinoma) and CT26 (mouse colorectal carcinoma) cells were purchased from ATCC and were propagated according to the supplier’s instructions. The murine recycled prostate cancer cell line TRAMPC1 was kindly provided by Dr. Richard Ciavarra of Eastern Virginia Medical School, Norfolk USA, and propagated as described in [21].

### *In Vitro* cytotoxicity assay

The 3-(4, 5-dimethylthiazol-2-yl)-2, 5-diphenyltetrazolium bromide (MTT) assay was optimised for use as an end-point analysis for experiments involving the treatment of CT26, RENCA and TRAMP cell lines with TNFα. Stocks of MTT were prepared by dissolving MTT powder in phosphate buffered saline at a concentration of 5 mg/ml, and stored at -20 ^o^C (protected from over-exposure to light). Cells were seeded in 500 μl of relevant growth medium at 4 x 10^4^ cells per well in a 24 well flat bottomed plate (Sarstedt) and allowed to grow for 24 h. Wells were treated with varying concentrations of murine TNFα (Sigma-Aldrich) or vehicle (sterile water) in triplicate. Blank wells without cells were also included to account for any background fluorescence. After 48 h, MTT was added to cells at a final concentration of 0.5 mg/ml and incubated for 90 min at 37 ^o^C, 5% CO_2_. All medium was then aspirated carefully and purple formazan crystals were dissolved upon addition of 150 μl DMSO per well. Each well was mixed with 70 μl each sample and transferred to a 96-well microtest plate (Sarstedt) and absorbance read at 570 nm on an Infinite 2000 spectrophotmetric plate reader (Tecan). An Excel add-in ED50V10-2 was used for calculating half maximal effective concentration of TNFα (ED50), calculated from absorbance readings.

### Plasmid construction and transformation

The mRNA sequence for *Mus musculus* tumour necrosis factor alpha (GenBank: BC137720.1) was retrieved from the NCBI database. The gene was then synthesized and ligated into a custom made version of plasmid pSF-OXB20-Br322 by Oxford Genetics Ltd, Oxford, UK, containing a low copy origin of replication, strong promoter, and kanamycin resistance marker (S1A Fig). An empty version of the plasmid was also supplied. The plasmids were then transformed into *E*. *coli* MG1655-p16lux (herein referred to as MG) [22] and selected for and maintained on LB and Kan_50_ + Em_300_ and incubated at 37 °C.

### *In vitro* cytokine expression

To confirm the expression of TNFα by the plasmid constructs in MG and the absence of expression in the equivalent empty constructs, each isolate was grown in 10 mL LB for 18 h at 37 °C with appropriate antibiotics. A Mouse TNFα Single Analyte ELISA Kit (Qiagen) was used to test for the presence of the cytokine in all transformed plasmid constructs in the total bacterial lysate, as per manufacturer’s instructions.

### Murine experiments

All animal procedures were performed according to the national ethical guidelines of the Health Products Regulatory Authority (HPRA). Protocols were approved by the University College Cork Animal Experimentation Ethics Committee (AERR #2010/003 and #2012/015). Health status of all mice was monitored daily for the duration of experiments. There were no deaths outside of humane euthanasia. Mice were humanely euthanized by cervical dislocation upon tumours reaching a size of 1.5 x 1.5 cm in diameter. To minimize suffering or distress during invasive procedures (imaging and bacterial injection), mice were anaesthetised with isoflourane (2.5% mixture with oxygen).

### Animals and tumour induction

Mice were kept at a constant room temperature (22 °C) with a natural day/night light cycle in a conventional animal colony. Standard laboratory food and water were provided ad libitum. Before experiments, the mice were afforded an adaptation period of at least 7 days. Male C57Bl (TRAMPC1 model) and female Balb/C (RENCA and CT26 models) mice in good condition, without fungal or other infections, weighing 16–22 g and of 6–8 weeks of age, were included in experiments (Harlan, Oxfordshire, UK). At experiment end, animals were euthanized by cervical dislocation. For routine tumour induction, the minimum tumorigenic dose of cells (5 x 10^5^ CT26, 1 x 10^5^ RENCA, 5 x 10^5^ TRAMPC1) suspended in 200 μl serum-free culture medium was injected subcutaneously (s.c.) into the flank. The viability of cells used for inoculation was greater than 95% as determined by the Nucleocounter system (ChemoMetec, Bioimages Ltd, Cavan, Ireland). Following tumour establishment, tumours were allowed to grow and develop and were monitored three times weekly. Tumour volume was calculated according to the formula V = (ab2) Π /6, where a is the longest diameter of the tumour and b is the longest diameter perpendicular to diameter a. When tumours reached approximately 100 mm^3^ in volume, mice were randomly divided into experimental groups.

### *In vivo* bacterial administration

Inocula were prepared by growing MG-Empty and MG-TNFα with or without the integrated p16Slux aerobically in 100 mL LB broth containing either 50 μg/mL Kan (MG1655) or 50 μg/mL + 300 μg/mL Em (MG1655p16Slux). Overnight cultures were re-inoculated into fresh LB (1/50 dilution) and incubated shaking at 37°C until they reached an OD600 of 0.6–0.8. Cultures were harvested by centrifugation (13,500 g for 1 min), washed three times with PBS and resuspended in a one tenth volume of PBS. Tumours were administered 10^6^ *E*. *coli* by either intratumoural (i.t.) injection (RENCA and TRAMPC1 model) or intravenous (i.v.) injection via the lateral tail vein (CT26 model). The viable count of each inoculum was determined by retrospective plating on LB agar containing the appropriate selective antibiotic.

### *In vivo* bioluminescence imaging (BLI)

At defined time-points after bacteria, animals were anesthetized by intraperitoneal administration of 200 mg xylazine and 2 mg ketamine and 2D *in vivo* Bioluminescence Imaging was performed using the IVIS Lumina II (Perkin Elmer, Waltham, MA) with 2 min integration times at high sensitivity. Following whole-body imaging, the mice were euthanized via cervical dislocation and the subcutaneous tumours were aseptically removed and imaged. For each experiment, images were captured under identical exposure, aperture and pixel binning settings, and bioluminescence is plotted on identical colour scales. Bioluminescent signal was quantified by creation of regions of interest (ROIs). To standardize the data, light emission was quantified from the same surface area (ROI) for each tumour. Imaging data was analysed and quantified with Living Image Software (Perkin Elmer) and expressed as photons/second/cm^2^.

### Bacterial recovery from the tumour

Following imaging, each tumour was aseptically cut into three sections, one-third of which was immediately homogenized by fine mincing using a scalpel and pushed through a 20 μm pore nylon filter (Falcon, Becton Dickinson (BD), Oxford, England). The filter was then rinsed with 2 mL of LB broth to create a cell suspension. Serial dilutions were plated in triplicate on LB agar containing selective antibiotics, grown overnight at 37°C and the resulting colonies used to calculate the number of bacterial cells per tissue sample.

### Immunofluorescence

One third of the tumour was snap frozen in optimal cutting temperature compound (Tissue-Tek; Sakura Finetek) using liquid nitrogen and isopentane. Frozen tumour sections (5 μm) were fixed in an ice-cold acetone-alcohol mixture (3:1 ratio), blocked with blocking serum for 45 min at RT in a humidified chamber, stained with purified rabbit polyclonal anti-*E*. *coli* antibody (Abcam, UK) and counterstained using donkey anti-rabbit Alexa Fluor 488-conjugated anti-Ig antibody (Jackson Immunoresearch Laboratories Inc., USA). Stained sections were mounted in ProLong Gold antifade reagent with DAPI (Invitrogen, UK) and visualized using a fluorescence microscope (Olympus BX51).

### Cytokine profiling

Snap frozen tumour sections were thawed, weighed and placed into Lysing Matrix A tubes (Mp Biomedicals, Medical Supply Company, Dublin, Ireland) containing 1 mL of homogenization buffer (50 mL PBS + 1 protease inhibitor cocktail tablets (Roche) + 10% FCS and homogenized using a FastPrep FP120 Cell disrupter (Qbiogene, Cedex, France). Homogenized samples were centrifuged for 12 min at 14,000 RPM at 4°C and the supernatants collected and stored at -80°C. Cytokine concentrations in the supernatants were measured by a Meso Scale Discovery 7-plex pro-inflammatory cytokine plate (MSD, Gaithersburg, MD, USA) according to the manufacturer’s instructions. The plates were analysed on the MSD Sector 2400 Imager (MSD).

### Statistical analysis

Statistical significance was determined with unpaired Student’s T test or Gehan-Breslow-Wilcoxon test for survival curves. All statistical tests were performed using commercially available statistic software (GraphPad Software, CA, USA). Data are represented by Mean ± (Standard Error of the Mean) SEM, unless otherwise stated. P values of < 0.05 were considered significant (*P < 0.05, **P < 0.01).

## Results

### *E*. *coli* growth in tumours

**Fig 1:**
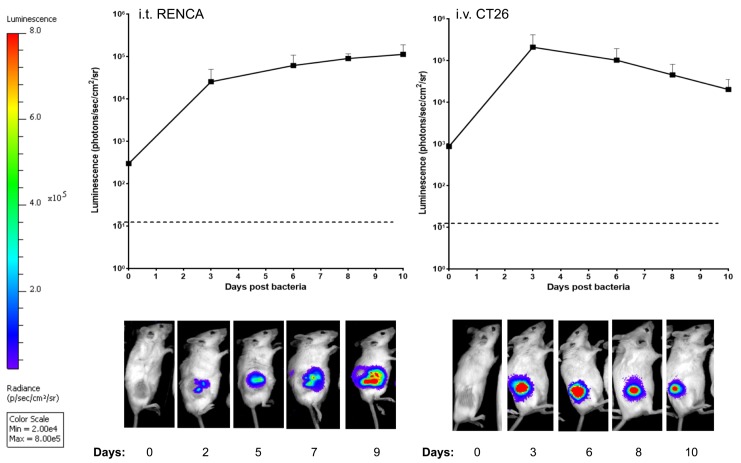
Administration of luminescent *E*. *coli* MG1655 to tumour bearing mice. Subcutaneous RENCA tumours were injected i.t. with 10^6^ cfu and monitored by BLI over the shown time points (left). Mice (n = 6) bearing CT26 tumours were injected with 10^6^ bacteria to the lateral tail vein and all mice monitored at each time point over the course of time (right). The change in bacterial luminescence (relative to day 0) is shown. A representative image at each time point is shown. Luminescence remained stable across the range of time-points indicating that robust numbers of viable bacteria persisted within the tumour throughout the experiment.

To validate the utility of this platform, a bioluminescent form of MG1655 carrying the lux cassette in its genome (herein referred to as MG) was employed, which has previously been validated in this setting in our laboratory [22]. MG was administered to Balb/C mice bearing RENCA tumours i.t or CT26 tumours i.v. and monitored by whole-body bioluminescence imaging (BLI) over time. Persistent bioluminescence signal was observed for >9 days indicating bacterial survival and growth in tumours (Fig 1).

### Engineered *E*. *coli* facilitates TNFα production within tumours

**Fig 2:**
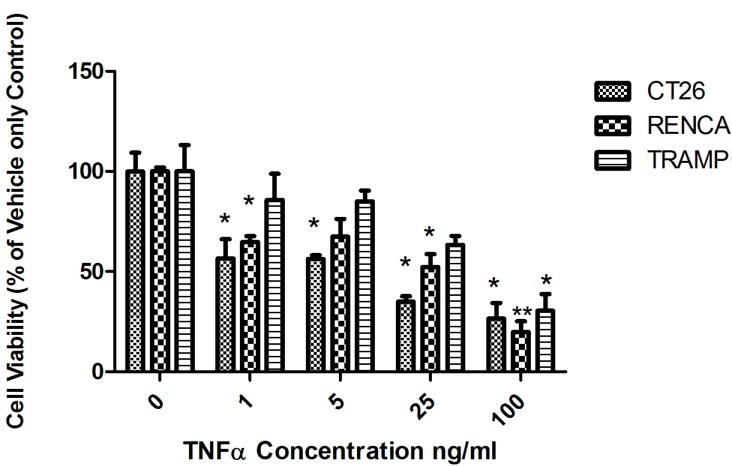
Tumour cell sensitivity to TNFα *in vitro*. MTT-based *in vitro* cytotoxicity assays following incubation of CT26, RENCA or TRAMP cells with varying concentrations of TNFα indicate significant sensitivity to TNFα with all cell lines examined (*p < 0.05, **p < 0.01).

A TNFα -expressing MG was designed and generated as described in Materials & Methods and S1 Fig. TNFα production from the construct was validated *in vitro* by ELISA (S1B Fig). *In vitro* cytotoxicity assays with the various cell lines in the presence of TNFα indicated significant sensitivity to TNFα with all cell lines examined; ED50 CT26–37 ng/ml, RENCA 50 ng/ml, TRAMP 64 ng/ml; (Fig 2).

**Fig 3:**
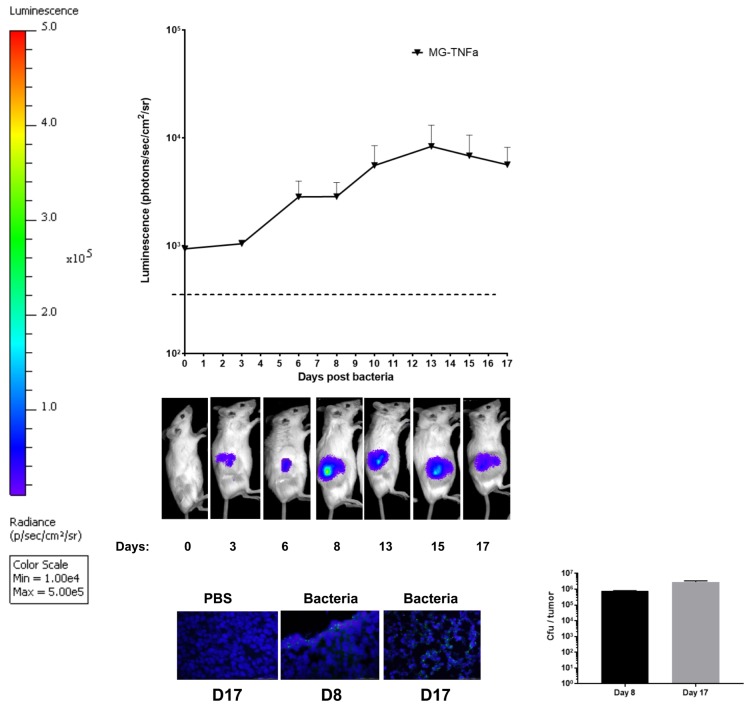
Intravenous administration of MG-Tnfα to tumour bearing mice. Balb/C mice bearing s.c. CT26 flank tumours (n = 6) received 10^6^ cfu of MG-TNFα i.v.. Growth of bacteria in tumours was analysed by **(a)** BLI and **(b)** immunofluorescence (IF), while **(c)** plating of tumour extracts on agar plates quantified viable bacteria. A representative image for each BLI group is shown. For IF, tissue sections from 2 individual mice per time point were analysed by fluorescence microscopy. (Original magnification, 400x), Scale bars, 50 μm.

**Fig 4:**
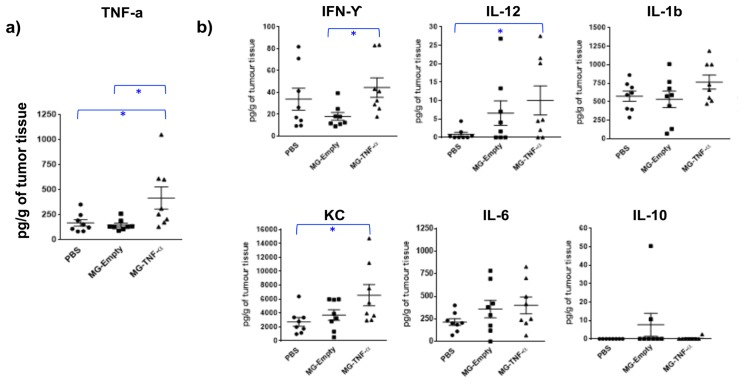
Intratumoural TNFα production and cytokine profiles. **(a)** Cytokine analysis of CT26 tumour extracts following treatment with bacteria. **(b)** Multiplex cytokine analysis of treated CT26 tumour extracts. * p ≤ 0.05.

In order to qualitatively assess tumour targeting and proliferation of MG-TNFα, bacteria were administered to Balb/C mice bearing CT26 tumours (i.v.) and monitored by BLI and immunofluorescence (IF) over time. BLI demonstrated increasing numbers of this strain within tumours for two weeks (Fig 3). IF specific for *E*. *coli* confirmed bacterial presence within tumour tissue, and viable bacteria were recovered at all-time points examined. ELISA analysis of tumour homogenates demonstrated significantly higher TNFα levels within tumours of MG-TNFα treated mice compared with controls (mice i.v. administered engineered MG1655 lacking the TNFα gene or PBS) (Fig 4A).

Induction of inflammatory cytokines within CT26 tumours was examined by MSD multiplex cytokine analysis of tumour homogenate. A pattern of pro-inflammatory cytokine induction was observed in tumours of MG-TNFα treated mice vs. controls (Fig 4B).

### *In vivo* therapy of prostate, colon and renal carcinoma

**Fig 5:**
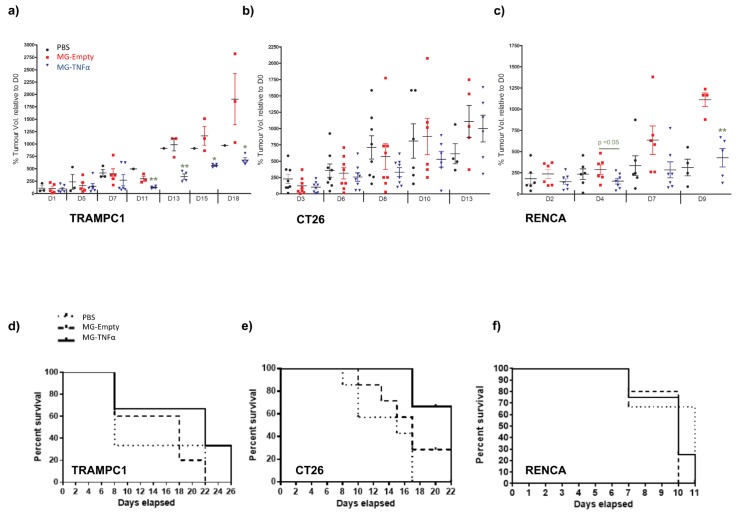
Tumour therapy via *in vivo* production of Tnfα. BALB/c or C57 mice bearing s.c. flank tumours were administered MG-Empty, MG-TNFα or vehicle (PBS). Tumours were monitored for changes in volume every other day. Tumour volume (%) relative to the first day of bacterial administration (day 0) is shown for (i) TRAMPC1 (ii) CT26 and (iii) RENCA **(a-c)**. Statistical analysis at each time point is based on number of subjects alive. **(d-e)** Kaplan-Meier survival plots of each group starting from the first day of bacterial administration. *p < 0.05, **p < 0.01.

Mice bearing subcutaneous flank tumours (TRAMPC1, CT26 and RENCA) were administered PBS, MG-Empty or MG-TNFα i.t (TRAMP, RENCA) or i.v. (CT26) and tumour volume and survival monitored over time (Fig 5). Therapeutic effects were observed in all studies, albeit at varying levels (Table 1). In the TRAMP prostatic tumour model, tumour growth was significantly reduced in MG-TNFα treated mice versus controls (p < 0.05, p < 0.01), but apparent increase in median survival was not significant compared with MG-Empty (p > 0.05). Median survival of mice bearing CT26 tumours treated with MG- TNFα was significantly increased vs. MG-Empty (p = 0.049), although the apparent reduction in tumour volume was not significant vs. controls (p >0.05). Significant reduction in tumour volume was observed at certain timepoints in the RENCA renal tumour model (p < 0.01), with no significant increase in survival (p > 0.05) in this rapid growing model. Mice remained healthy throughout experiments as evidenced by a disease activity index (S1 Table) [23] which was constructed by frequent monitoring of behaviour and appearance.

**Table 1:** Tumour growth responses to therapy.

	Tumour Growth (Volume)	Survival
**TRAMP (i.t.)**	Reduction significant	Increase not significant
**CT26 (i.v.)**	Reduction not significant	Increase significant
**RENCA (i.t.)**	Reduction significant	No increase

## Discussion

To our knowledge, this is the first *in vivo* study that demonstrates TNFα delivery by bacteria to experimental murine tumours. *E*. *coli* MG1655 was employed to deliver and produce the therapeutic biomolecule TNFα inside murine tumours. Initially, both i.t. and i.v. routes of administration were tested to compare tumour targeting, and MG1655 performed well in both. For more clinical potential (for inaccessible and metastatic cancers), the i.v. route was chosen and MG-TNFα was administered to mice bearing CT26 tumours for further qualitative and quantitative analysis. MG-TNFα was capable of targeting tumours and proliferating as evidenced by BLI, IF and cfu counts. TNFα production within CT26 tumours was confirmed by ELISA and therapeutic studies indicated that MG-TNFα can impede tumour growth without inducing significant systemic toxicity.

TNFα is a cytokine that is highly toxic to cells and therefore has been selected as a therapeutic biomolecule for this study. In the past, genetic constructs for *in vitro* production of TNFα by *Clostridium* were described [19, 24] but therapeutic analyses have not been reported to our knowledge. MG1655 was chosen because it is a strain that has been very well characterized by the scientific community, is relatively safe compared with other vectors employed and is easy to engineer. MG1655 handles overexpression of proteins and engineered transcriptional regulation well (e.g. [25]) making it overall a suitable platform for *in situ* production of therapeutic biomolecules. Bacteria were tolerated well by mice in all experiments without any observable toxicity. The lux imaging system employed provided a robust method to track bacterial viability (Figs 1 and 3). We have reported the employment of this method for *E*. *coli* MG1655 for colonising murine tumours several times [22, 26–29], including studies directly correlating culture enumeration with lux imaging as a quantitative and spatial readout for bacterial growth over time. For example, we have previously reported an R^2^ value of 0.97 in relationship between subcutaneous tumour MG1655 numbers and lux bioluminescence [22].

MG-TNFα administered i.v. was able to reach its maximum level within a few days and persisted in tumours for approximately two weeks. Similar tumour targeting dynamics have previously been reported with various bacteria [22, 26, 30]. IF studies revealed *E*. *coli* within tumour tissue of treated mice only. TNFα production was achieved using MG-TNFα as evidenced by analysis of tumour extracts. Immune competent syngeneic mice were utilised for all studies, and downstream local pro-inflammatory effects were apparent following treatment as evidenced by cytokine analysis, suggesting local secretion by immune cells following treatment. In terms of immune responses to the bacterial vehicle, Fig 4B indicates that the bacterial backbone (MG–Empty) induced a degree of immune response as evidenced by an increase in a number of cytokines (IL-12, IL-6 and IL-10), although the increases over PBS are not statistically significant (p = 0.108, p = 0.199, p = 0.242 respectively). Furthermore, in this study, lux (= > live *E*. *coli*) was observed for the duration of the experiment (Fig 1), although i.t. administration provided stable luminescence for 10 days, while i.v. luminescence reduced slightly over time, indicating a reduction in bacterial numbers, possibly related to an immune response against systemically administered bacteria, more so than locally administered bacteria. These findings (in immune-competent syngeneic Balb/C mice) contrast with our previous observations in immune-reduced athymic mice (MF1nu/nu), where we observed no increase in pro-inflammatory cytokines following IV administration of MG1655 [26]. The delicate balance of inflammatory and suppressive cytokines with different tumour models may be affected by the empty vector, and may also vary between tumour models and stages of tumour growth. There is also potential that some therapeutic effects of the TNFα produced may be masked due to opposing responses to the bacterial vehicle–e.g. IFNγ was reduced in MG-Empty treated mice compared with PBS.

The field of engineered bacterial cancer therapy is advancing at a fast pace. Since its infancy 20 years ago, many systems have been tested providing feedback for better strategic decisions regarding payload choice and vector design [31]. We chose to employ a highly toxic payload with documented clinical use that can be locally produced *in situ* by a non-toxic cancer targeting vehicle. Further modifications are possible; for example, sophisticated synthetic biology has allowed for state of the art regulation of protein production and delivery to tumours maximizing therapeutic efficacy [32]. The ability to control expression of highly toxic TNFα via exogenous induction [33] or a self-regulating circuit [32] in an improved iteration would make such a strategy more attractive for clinical development from a safety perspective.

Overall, MG1655 is a strain that responds well to artificial transcriptional regulation and tolerates protein over-production. Future work may involve advanced synthetic biology techniques to further improve our vehicle and payloads on many levels; from the bacterial ‘chassis’ to ‘device’-mediated *in situ* production. Bacterial production of therapeutic biomolecules for cancer therapy is now looking more promising than ever.

## Supporting information

S1 Fig Engineered strain construction and validation. **(a)** Plasmid Construct **(b)** ELISA data for TNFα production from MG extracts *in vitro*. **(c)** *In vitro* growth rates of TNFα -producing bacteria, indicating no significant toxic effects on the host bacterium.
(EPS)

S1 Table Toxicity/ dose response study. 6–8 week old tumour-free BALB/c mice (n = 3 per group) were administered a high (10^7^cfu) or low (10^6^ cfu) dose of MG-TNFα via i.v. injection to the tail vein. Mice were monitored at regular intervals post bacteria for the following macroscopic health indicators; Fur texture (0 smooth coat; 1 mildly scruffy; 2 very hunched); Posture (0 not hunched; 1 mildly hunched; 2 very hunched); and Activity (0 active; 1 less active than normal, 2 inactive) to create a disease activity index.
(DOCX)
