# Supplementary material for: Intratumoural production of TNFα by bacteria mediates cancer therapy
Source: PLoS One. 2017 Jun 29;12(6):e0180034. doi: 10.1371/journal.pone.0180034 (PMC5491124; doi:10.1371/journal.pone.0180034)
Supplement: S1 Table — 6–8 week old tumour-free BALB/c mice (n = 3 per group) were administered a high (107cfu) or low (106 cfu) dose of MG-TNFα via i.v. injection to the tail vein. Mice were monitored at regular intervals post bacteria for the following macroscopic health indicators; Fur texture (0 smooth coat; 1 mildly scruffy; 2 very hunched); Posture (0 not hunched; 1 mildly hunched; 2 very hunched); and Activity (0 active; 1 less active than normal, 2 inactive) to create a disease activity index. (DOCX) [file pone.0180034.s002.docx]

**DAI (Disease Activity Index)** 0 = Normal, 1 = Slightly, 2 = Moderately, 3 = Very

|  | **Posture** | **Activity** | **Ruffled fur** |
| --- | --- | --- | --- |
| **High Dose (1 h post i.v.)** | 0 | 0 | 1 |
| **Low Dose (1 h post i.v.)** | 0 | 0 | 0 |
| **High Dose (17 h post i.v.)** | 0 | 0 | 1 |
| **Low Dose (17 h post i.v.)** | 0 | 0 | 0 |
| **High Dose (48 h post i.v.)** | 0 | 0 | 1 |
| **Low Dose (48 h post i.v.)** | 0 | 0 | 0 |
| **High Dose (72 h post i.v.)** | 0 | 0 | 0 |
| **Low Dose (72 h post i.v.)** | 0 | 0 | 0 |
| **High Dose (144 h post i.v.)** | 0 | 0 | 0 |
| **Low Dose (144 h post i.v.)** | 0 | 0 | 0 |
| **High Dose (240 h post i.v.)** | 0 | 0 | 0 |
| **Low Dose (240 h post i.v.)** | 0 | 0 | 0 |
